# Editorial: Wildlife-related zoonotic infections: major threat to public health

**DOI:** 10.3389/fvets.2024.1532732

**Published:** 2024-12-16

**Authors:** Jelena Prpić, Fábio A. Abade Dos Santos, Magdalena Petrova Baymakova

**Affiliations:** ^1^Department of Virology, Croatian Veterinary Institute, Zagreb, Croatia; ^2^Faculty of Veterinary Medicine, Lusofona University, Lisbon, Portugal; ^3^National Institute for Agricultural and Veterinary Research (INIAV), Oeiras, Portugal; ^4^Department of Infectious Diseases, Military Medical Academy, Sofia, Bulgaria

**Keywords:** bacteria, disease control and prevention, One Health, parasites, public health, surveillance, viruses, wildlife diseases

## Introduction

The emergence of zoonotic diseases, particularly those originating from wildlife, poses a significant threat to global public health (1). These infections, which are transmitted from animals to humans, account for ~75% of all emerging infectious diseases, with 70% of these originating from wildlife (1–5). The increasing frequency and impact of these diseases underscore the urgent need for comprehensive research and proactive measures to mitigate their spread.

## Aims and objectives

The primary aim of this editorial is to highlight the critical research contributions that address the complex dynamics of wildlife-related zoonotic infections. By examining various studies, we aim to provide a broader context for understanding the mechanisms driving these infections and the strategies necessary for their prevention and control.

## Broader context

The interaction between wildlife, domestic animals, and humans creates a complex web of pathogen transmission. Human activities such as deforestation, urbanization, and agricultural expansion disrupt natural habitats, increase the likelihood of zoonotic spillover events. The COVID-19 pandemic has starkly illustrated the devastating potential of zoonotic diseases, emphasizing the need for a One Health approach that integrates human, animal, and environmental health.

## Key research contributions

The contribution from Roque et al. provides a valuable perspective regarding the biodiversity and land use change. The study explores how changes in land use impact biodiversity and the emergence of zoonotic diseases. The research underscores the importance of preserving biodiversity to prevent the emergence of new zoonotic pathogens. It highlights the need to incorporate biodiversity responses into land use change scenarios to prevent emerging zoonotic diseases in areas with unknown host-pathogen interactions. Incorporating biodiversity responses to land use change scenarios for preventing emerging zoonotic diseases in areas of unknown host-pathogen interactions. The contribution from Kabzhanova et al. provides a detailed analysis of rabies spread in Kazakhstan. This study highlights the spatial and temporal patterns of rabies, offering insights into effective control measures. The research done by Azat et al. examines the factors driving the spread of avian influenza. This study is crucial for understanding the spatio-temporal dynamics and drivers of highly pathogenic avian influenza H5N1 in Chile, and for developing strategies to mitigate its impact. The prevalence and diversity of Blastocystis in wild rodent populations was investigated in the study done by Gao et al. This research provides valuable data on the potential zoonotic risk posed by wild rodents. It examines the prevalence and subtypes of Blastocystis in wild rodents from three provinces in China. Lienen et al. explored the toxinogenic potential and antibiotic resistance of *Staphylococcus aureus* in wild ungulates. This study highlights the public health risks associated with wildlife reservoirs of antibiotic-resistant bacteria and the high toxinogenic potential of *Staphylococcus aureus* from wild ungulates in Brandenburg, Germany with a low level of antibiotic resistance. Lukina-Gronskaya et al. provide insights into the viral diversity in hedgehogs and the potential zoonotic threats they pose. This study emphasizes the need for continuous surveillance of wildlife to detect emerging pathogens early.

## Conclusion

The studies highlighted in this editorial underscore the critical need for a multidisciplinary approach to understanding and mitigating wildlife-related zoonotic infections. By integrating ecological, epidemiological, and molecular research, we can develop more effective strategies to predict, prevent, and control these diseases. The One Health approach, which recognizes the interconnectedness of human, animal, and environmental health, is essential for addressing the complex challenges posed by zoonotic diseases.

As we continue to encroach on natural habitats and alter ecosystems, the risk of zoonotic spillover events will likely increase. Therefore, it is imperative to invest in research, surveillance, and public health infrastructure to safeguard against future outbreaks. The contributions to this Research Topic provide valuable insights and pave the way for future studies aimed at protecting global health from the threat of wildlife-related zoonotic infections.

## References

[1] Kruse H. Wildlife as source of zoonotic infections. Emerg Infect Dis J. (2004) 10:2067–72. 10.3201/eid1012.040707 15663840 PMC3323390

[2] World Health Organization. WHO Consultation on Public Health and Animal Transmissible Spongiform Encephalopathies: Epidemiology, Risk and Research Requirements. Geneva (1999). Available at: https://apps.who.int/iris/handle/10665/66422 (accessed February 25, 2023).

[3] Weiss S Nowak K Fahr J Wibbelt G Mombouli JV Parra HJ. Henipavirus-related sequences in fruit bat bushmeat, Republic of Congo. Emerg Infect Dis. (2012) 18:1527–36. 10.3201/eid1809.111607 22935105 PMC3437727

[4] Pernet O Schneider BS Beaty SM LeBreton M Yun TE Park A. Evidence for henipavirus spillover into human populations in Africa. Nat Commun. (2014) 5:5342. 10.1038/ncomms6342 25405640 PMC4237230

[5] Taylor LH Latham SM Mark E. Risk factors for human disease emergence. Philos Trans R Soc Lond Ser B Biol Sci. (2001) 356:983–9. 10.1098/rstb.2001.0888 11516376 PMC1088493

